# Development of a Subjective Symptom Rating Scale for Postoperative Oral Dysfunction in Patients with Oral Cancer: Reliability and Validity of the Postoperative Oral Dysfunction Scale-10

**DOI:** 10.3390/diagnostics11112061

**Published:** 2021-11-07

**Authors:** Yuhei Matsuda, Isami Kumakura, Tatsuo Okui, Masaaki Karino, Noriaki Aoi, Satoe Okuma, Mayu Takeda, Kenji Hayashida, Tatsunori Sakamoto, Takahiro Kanno

**Affiliations:** 1 Department of Oral and Maxillofacial Surgery, Shimane University Faculty of Medicine, Izumo 693-8501, Japan; kumakurakobe@gmail.com (I.K.); tokui@med.shimane-u.ac.jp (T.O.); karino71@med.shimane-u.ac.jp (M.K.); okuma125@med.shimane-u.ac.jp (S.O.); mtakeda@med.shimane-u.ac.jp (M.T.); tkanno@med.shimane-u.ac.jp (T.K.); 2 Department of Otolaryngology, Shimane University Faculty of Medicine, Izumo 693-8501, Japan; nori-aoi@med.shimane-u.ac.jp (N.A.); sakamoto_tatsunori@med.shimane-u.ac.jp (T.S.); 3 Department of Plastic and Reconstructive Surgery, Shimane University Hospital, Izumo 693-8501, Japan; kenji@med.shimane-u.ac.jp

**Keywords:** postoperative oral dysfunction scale-10, oral cancer, oral function, oral dysfunction, dysphagia, reliability, validity

## Abstract

Currently, there is no scale to subjectively assess postoperative oral dysfunction in patients with oral cancer. The purpose of this study was to evaluate the reliability and validity of the Postoperative Oral Dysfunction Scale (POD-10) that we developed. Between September 2019 and August 2021, 62 eligible oral cancer patients (median age, 72 years; 42 men and 20 women) were enrolled in the study. The Cronbach’s alpha coefficient, which indicates the internal consistency of the scale, was 0.94, and the intraclass correlation coefficient, which indicates reproducibility, was 0.85 (95% confidential interval: 0.40–0.96, *p* < 0.05). Concurrent validity testing showed a statistically significant correlation between POD-10 and Eating Assessment Tool (EAT-10) (r = 0.89, *p* < 0.05). To test discriminant validity, statistically significant differences were found between early-stage cancer (stage I and II) and advanced-stage cancer (stage III and IV) (*p* < 0.05). Twenty-four points were calculated as the cutoff value for POD-10 using receiver operating characteristic analysis to calculate the cutoff value. The POD-10 was shown to be a clinically reliable and valid scale that can be used to subjectively assess postoperative oral dysfunction in patients with oral cancer and is expected to be used as a simple diagnostic tool.

## 1. Introduction

Adjuvant therapies (e.g., novel chemotherapy with immune checkpoint inhibitors and radiotherapy with intensity-modulated radiation therapy) have been developed for the treatment of oral cancer [1,2]. However, the fact that surgical treatment is still the first choice for oral cancer treatment remains unchanged in various guidelines [3]. Postoperative oral dysfunction occurs in many cases after the surgical treatment of oral cancer [4]. In our previous report, we reported that postoperative oral dysfunction can be diagnosed by a comprehensive evaluation of six areas (microorganisms, oral dryness, occlusal force, tongue pressure, masticatory function, and Eating Assessment Tool [EAT-10]) and that postoperative oral dysfunction can be divided into three types, according to Matsuda-Kanno (MK) classification (Type I, transport type; Type II, occlusion type; Type III, oral hygiene type; Table 1) [4]. These three types of classification include symptom assessment of subjective oral function using the visual analogue scale, which reflects the fact that patient-reported outcomes version of the Common Terminology Criteria for Adverse Events and quality of life assessment play an important role in the evaluation of oral cancer treatment [5,6].

On the other hand, the EAT-10, which has been adopted to assess oral function, is a subjective rating scale. In the report by Belafsky et al. on the development of EAT-10, 18% of the eligible patients had head and neck cancer, but it is unclear how many of these patients had oral cancer [7]. In addition, results of a randomized controlled trial examining the association of EAT-10 with fiberoptic endoscopic evaluation of swallowing findings (9 [16%] of 57 head and neck patients with oral cancer) suggested that EAT-10 is a strong reflection of swallowing status, primarily in the pharyngeal phase [8]. Therefore, the EAT-10 may have an inferior response in assessing oral stage disorder as a swallowing function in patients undergoing oral cancer surgical treatment, where oral dysfunction occurs due to organic changes in the oral cavity.

In fact, previous studies have shown that patients who have undergone surgical treatment for oral cancer experience problems specific to oral stage disorders [9]. If the surgical site extends around the gingiva, it has been reported that maxilla and mandibular loss may occur, and masticatory function may be impaired [10]. It has been reported that tongue treatment increases food residue in the oral cavity [11]. Oral morphological changes are known to cause sialorrhea by reducing the ability of the mouth to retain saliva [12]. It is also widely known that resection of the tongue results in organic articulation disorders, which makes it difficult to improve function to the preoperative level because it is caused by organic changes [13]. In addition, morphological changes in the perioral region have been reported to affect not only the esthetic but also the social and psychological aspects of patients [14]. These disorders are especially those that occur when the treatment area extends to the oral cavity and are different from those caused by pharyngeal surgery in head and neck cancer. Therefore, there is a need for a specific scale to assess postoperative oral dysfunction in patients treated for oral cancer.

The purpose of this study was to examine the reliability and validity of our novel scale developed for the subjective assessment of POD-10 after oral cancer treatment.

## 2. Patients and Methods

### 2.1. Data Collection

#### 2.1.1. Patients

Eligible patients were enrolled after confirming that they met the following criteria for inclusion: (1) patients who received treatment for oral cancer; (2) patients who visited the Department of Oral and Maxillofacial Surgery/Oral Care Center, Shimane University Hospital (Izumo Shimane, Japan); (3) adults 20 years of age or older; and (4) those who were able to complete the questionnaire on their own initiative. No exclusion criteria were established, and data were collected after completion of all primary treatments. The study period was from September 2019 to August 2021. Sixty-two patients were enrolled as eligible patients using the sequential sampling method. This study was conducted with the permission of the Institutional Review Board of the Ethics Committee of Shimane University Faculty of Medicine (number 4041). All eligible patients were also enrolled in the study after explanation, and written informed consent was obtained.

#### 2.1.2. Background Data

We cross-sectionally collected the following information from electronic medical records and questionnaires: age (years), sex (male/female), body mass index (BMI, kg/m^2^), Brinkman index, drinking habit (none, social drinker, or regular drinker), Eastern Cooperative Oncology Group Performance Status (PS), primary tumor site (tongue, maxillary gingiva, mandibular gingiva, palate, oral floor, buccal mucosa, central mandible, and lower lip), cancer stage based on the criteria of the Union for International Cancer Control (version 8), method of treatment (surgery, surgery and radiotherapy, surgery and chemotherapy, and surgery and chemoradiotherapy), presence of neck dissection and reconstructive surgery, number of teeth, functional oral intake scale (FOIS), repetitive saliva swallowing test (RSST), and Mini Nutritional Assessment-Short Form (MNA-SF).

#### 2.1.3. Oral Function Measurement

Oral function measurement was performed using the following medical devices, and measurements and data collection were performed in accordance with the specified measurement methods: measuring the number of oral microorganisms using a bacterial counter (DU-AA01NP-H, Panasonic Healthcare Co., Ltd., Tokyo, Japan), measuring the oral dryness using an oral moisture checker (Mucus, Life Co., Ltd., Saitama, Japan), measuring the occlusal force using a pressure-indicating film (Dental Prescale Occluzer, GC Co., Tokyo, Japan), measuring the tongue pressure using a tongue pressure measuring instrument (JMS tongue pressure measuring instrument TPM-01, JMS Co., Ltd., Tokyo, Japan), measuring the masticatory function using a masticatory ability testing system (Gluco Sensor GS- II, GC Corporation, Tokyo, Japan), and measuring the swallowing function using EAT-10 (developed by Belafsky et al., 2008).

Microorganisms were sampled for the grade values calculated using the device. Specimens were collected from the remaining healthy tongue if the tongue had been operated on or from the center of the flap from reconstructive surgery if total or subtotal glossectomy had been performed.

For oral dryness, the water content of the remaining healthy tongue was measured if the tongue had been operated on, and the water content of the center of the flap from reconstructive surgery was measured if total or subtotal glossectomy had been performed.

Occlusal force was measured with dentures, if available.

#### 2.1.4. Postoperative Oral Dysfunction Classification (MK Classification)

Postoperative oral dysfunction was assessed based on the classification of previous studies reported by our research team (MK classification; Table 1).

#### 2.1.5. Postoperative Oral Dysfunction Scale-10

**Figure 1:**
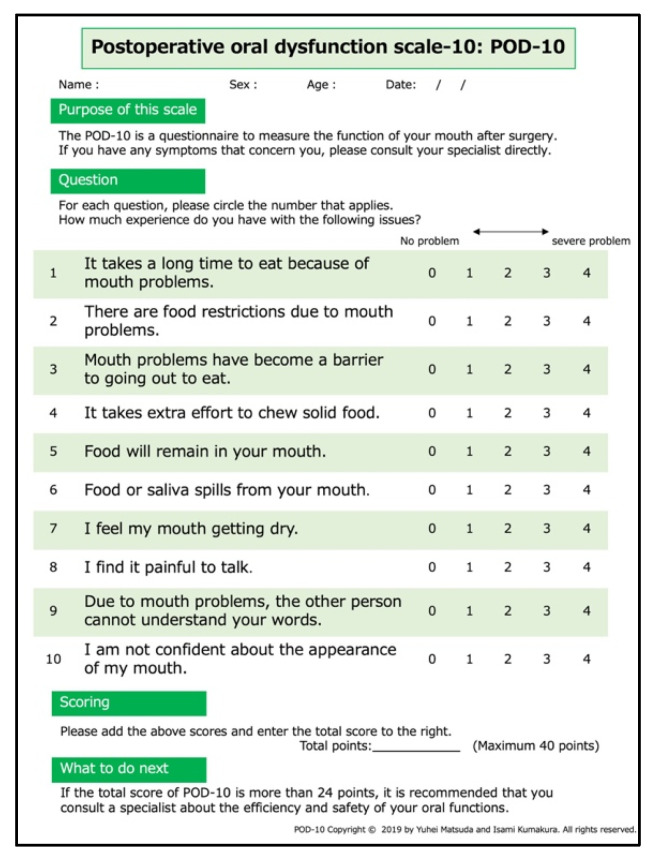
Japanese version of the postoperative oral dysfunction scale-10. The original scale is in Japanese. This English version of POD-10 has not been validated.

The POD-10 (Figure 1) was developed by oral surgeons, dental hygienists, and speech-language-hearing therapists with content validity. The POD-10 consists of 10 questions about postoperative oral dysfunction. A five-point Likert scale (0 = no problems to 4 = severe problems) was used to answer the questions. The higher the total score, the worse the oral function. To verify the reproducibility of the POD-10, a questionnaire survey was conducted twice on 10 randomly sampled participants.

### 2.2. Statistical Analysis

The normality test was determined using the Shapiro–Wilk test and was expressed as mean (standard deviation, SD) for parametric data, median (IQR) for non-parametric data, and number (%) for qualitative data. The ceiling effect was determined when the mean plus one SD exceeded the maximum value of the measurement, and the floor effect was determined when the mean minus one SD exceeded the minimum value of the measurement. The internal consistency of the POD-10 was evaluated by calculating Cronbach’s alpha coefficient. Reproducibility was evaluated by calculating the intraclass correlation coefficient (ICC) under the assumption of normality. Concurrent validity was assessed by calculating the Pearson’s correlation coefficient between the total score of the EAT-10 and the total score of POD-10. Discriminant validity was evaluated using a *t*-test as good–poor analysis based on the grouping method (comparison of early-stage cancer and advanced-stage cancer), where a significant difference was expected in the advanced stage predictions in FOIS (two groups of grades 6 and 7 and grades 1–5) and MNA-SF (two groups of normal nutritional status and at risk of malnutrition or malnutrition status) were evaluated by receiver operating characteristic (ROC) analysis as variables to guide the calculation of the cutoff value for POD-10. The area under the curve (AUC) and its 95% confidence interval were also calculated, and the point at which the AUC value was the maximum was adopted as the cutoff value.

Statistical analyses were performed using SPSS version 26 (SPSS Japan Inc., Tokyo, Japan). We calculated the two-tailed *p*-values for all analyses, and the alpha level of significance was set at *p* < 0.05.

## 3. Results

### 3.1. Patient Characteristics

The details of the patient characteristics are shown in Table 2. Sixty-two eligible patients (42 men [67.7%] and 20 women [32.3%]) were enrolled in this study. The mean age was 71.0 (IQR: 63.0–76.5) years. The PS of the 46 patients was 0 (74.2%). The most frequent primary tumor site was the tongue (n = 25, 40.3%). Early-tumor stage was observed in 20 patients (32.3%), and advanced-tumor stage was observed in 42 patients (67.7%). The most frequent method of treatment was surgery alone (n = 25, 40.3%). Neck dissection was performed in 42 patients (67.7%). Reconstruction surgery was performed in 40 patients (64.5%). The median number of teeth was 17.0 (IQR: 0.0–25.0). The median (IQR) values of oral function measurements were 4.0 (2.0–5.0), 24.6 (21.2–26.7), 270.4 (27.8–458.6), 15.7 (5.3–25.0), 58.0 (12.0–159.0), and 16.5 (5.5–25.3) for microorganisms (Grade), oral dryness, occlusal force (N), tongue pressure (kPa), masticatory function (mg/dL), and EAT-10, respectively. The median FOIS was 5.0 (IQR: 5.0–6.0). The median RSST was 3.0 (IQR: 3.0–4.0). The MNA-SF included 13 patients (21.0%), 25 patients (40.3%), and 24 patients (38.7%) for the normal nutritional status, at the risk of malnutrition status, and malnutrition status, respectively. Seventeen patients (27.4%) had no postoperative oral dysfunction, while 45 patients (72.6%) had postoperative oral dysfunction.

### 3.2. Descriptive Statistics of Postoperative Oral Dysfunction Scale-10 and Ceiling and Floor Effect

The descriptive statistics of the POD-10 are summarized in Table 3. The mean plus/minus one SD did not exceed the maximum or minimum score of the total POD-10 score.

### 3.3. Reliability

#### 3.3.1. Internal Consistency

The Cronbach’s alpha of the POD-10, consisting of 10 questions, was 0.94.

#### 3.3.2. Reproducibility

The ICC of POD-10 measured twice was 0.85 (95% confidence interval [CI]: 0.40–0.96, *p* < 0.05).

### 3.4. Concurrent Validity

**Figure 2:**
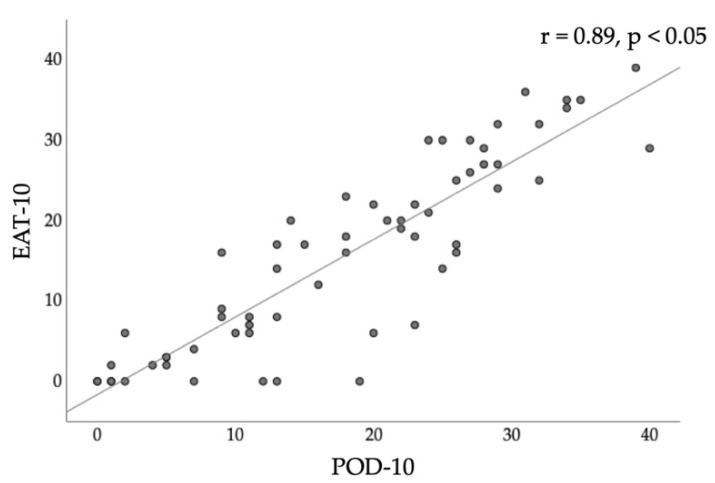
Scatter plots and linear correlations in total scores of POD-10 and EAT-10. EAT-10: eating assessment tool-10, POD-10: postoperative oral dysfunction scale-10.

The total scores on the POD-10 and EAT-10 showed a statistically significant correlation with Pearson’s correlation coefficient (r = 0.89, *p* < 0.05; Figure 2).

### 3.5. Discriminant Validity

**Figure 3:**
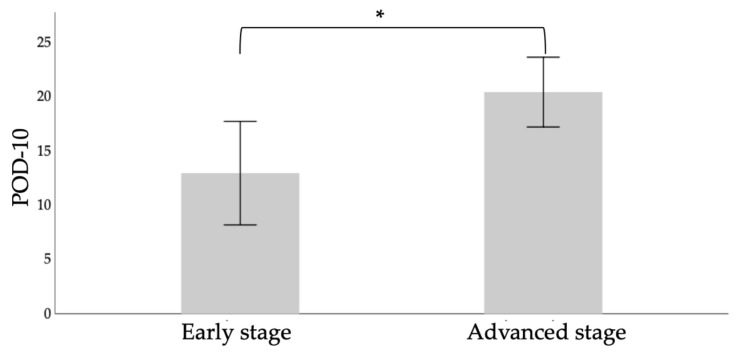
Dynamite plot comparing POD-10 of early-stage and advanced-stage cancers by *t*-test. POD-10: postoperative oral dysfunction scale-10. Error bar: 95% confidence interval, *: *p* < 0.05.

In the comparison by *t*-test between early-stage cancer (stage I and II) and advanced- stage cancer (stage III and IV), where a significant difference was expected beforehand, the total score of POD-10 showed a statistically significant difference (*p* < 0.05, Figure 3).

### 3.6. Cut-Off Value

**Figure 4:**
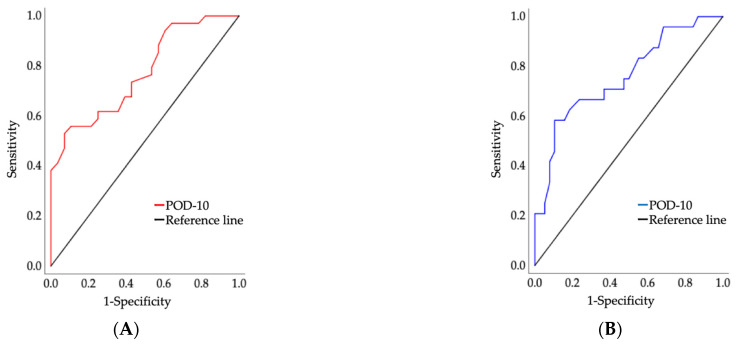
(**A**) Figure showing the AUC by ROC analysis to discriminate between the high-FOIS and low-FOIS groups; (**B**) Figure showing the AUC by ROC analysis to discriminate between the high-MNA-SF and MNA-SF groups. POD-10: postoperative oral dysfunction scale-10.

Receiver operating characteristic analysis was performed to investigate the accuracy of POD-10 in predicting FOIS (Figure 4A) and MNA-SF (Figure 4B). In predicting FOIS, results indicated the following: AUC of 0.77 (95% CI, 0.66–0.89; *p* < 0.05; cut-off value: 24.5 [sensitivity, 0.53; specificity, 0.93]) for POD-10. In predicting MNA-SF, the results indicated the following: AUC of 0.76 (95% CI, 0.63–0.88; *p* < 0.05; cut-off value: 25.5 [sensitivity, 0.58; specificity, 0.90]) for POD-10.

## 4. Discussion

This study had two main findings. First, this study provided the world’s first descriptive epidemiological information on postoperative oral dysfunction (three types and their combined types) in patients with oral cancer. Second, we developed a new scale (POD-10) to subjectively assess postoperative oral dysfunction after oral cancer treatment and proved its reliability and validity.

Postoperative oral dysfunction was the first disease name defined in our previous study [4]. Therefore, its prevalence and individual pathogenesis remain unclear. The results of this study showed that approximately 30% of patients had some form of oral dysfunction, and approximately 20% of cases were stage I, indicating that even early-stage cancer can cause oral dysfunction. In a similar previous study that reported the stages of oral cancer in Japan, 20% were stage I, 34% were stage II, 17% were stage III, and 29% were stage IV. The results of this study suggested that the incidence of oral dysfunction was also higher because of the greater severity of the cancer stage [15]. In the present study, the majority of postoperative oral dysfunction was Type II, but this may have been influenced by the fact that maxillary and mandibular gingival cancers together accounted for approximately 40% of the cases. On the other hand, postoperative oral dysfunction related to Type 1 was limited to approximately 20% of the total, suggesting that food transport disorders may be less likely to occur in patients with early-stage tongue cancer. In addition, it has been reported that swallowing function is affected by the TNM stage, particularly the T factor, and age [16,17]. From the above, it can be inferred that the degree of postoperative oral dysfunction is associated with the primary site and stage of the cancer, especially the T factor. A detailed analysis of each type is required in future studies.

Given that POD-10 performed well in terms of reliability and validation, it is considered clinically applicable. For ceiling and floor effects, both are generally defined as the mean score plus or minus one SD, not exceeding the highest or lowest score [18]. The mean score plus or minus one SD of POD-10 did not exceed the minimum and maximum total scores of POD-10; thus, no ceiling or floor effects were observed. However, it is important to consider the possibility that some patients may clinically complain of symptoms beyond those rated on the 5-point Likert scale. Cronbach’s alpha coefficient, which indicates internal consistency among reliability, was within the standard range of 0.70–0.95, but some reports suggest that 0.90 should be the standard, suggesting that the obtained value in this study may be too high [19,20]. According to the criteria of previous studies, ICC is defined as poor when it is less than 0.5, moderate between 0.5 and 0.75, good between 0.75 and 0.90, and excellent when it is greater than 0.90 [21]. Because the ICC of POD-10 was 0.85, it has good reliability and reproducibility. For Pearson’s correlation coefficient between POD-10 and EAT-10, which is used to test concurrent validity, previous studies on correlation coefficients have defined values below 0.2 as almost negligible relationships, 0.20–0.40 as weak, 0.40–0.70 as moderate, 0.70–0.90 as strong, and 0.90–1.00 as very strong [22]. The correlation coefficient between POD-10 and EAT-10 was 0.89, indicating that there is a strong correlation; therefore, the concurrent validity of POD-10 is considered to be good. In terms of discriminant validity using good–poor analysis, POD-10 is considered to have superior discriminant validity because statistically significant differences were detected in the groupings (early-stage and advanced-stage cancers) that were predicted to have significant differences in advance [23]. For the calculation of the cut-off value by ROC analysis, statistically significant AUCs were obtained for both the FOIS-based and MNA-SF-based methods, thus providing a significant predictive value. The cut-off values for the maximum AUC were 24.5 for FOIS and 25.5 for MNA-SF, respectively, but both had problems with low sensitivity. Hence, to increase sensitivity as much as possible, a cutoff value of 24 points was adopted, but considering actual clinical applications, a lower cutoff value should be considered. From the above, the POD-10 is considered to be a reliable, valid, and clinically applicable instrument for measuring postoperative oral dysfunction in patients undergoing oral cancer surgery.

Regarding the adaptation of POD-10 and EAT-10, POD-10 was developed with reference to EAT-10, but its application and the concept it measures are different. Therefore, the scale should be selected according to what one wants to measure in patients with oral cancer. In fact, in addition to the FOIS, the penetration-aspiration Scale is often used to evaluate the performance of the EAT-10, suggesting that the scale is primarily focused on the pharyngeal phase of swallowing or the Stage II transport-to-swallowing phase in the process model of swallowing [24,25]. However, considering that in the actual treatment of advanced oral cancer, neck dissection due to cervical lymph node metastasis, radiation therapy, and chemotherapy are performed as adjuvant therapy, pharyngeal stage disorders often occur even in oral cancer [26,27,28]. Therefore, POD-10 and EAT-10 should be used together in both research and clinical practice because both can be assessed with as few as 10 questions in patients with oral cancer.

There are three limitations to this study. First, among the background factors of the study participants, there may be a problem with generalizability because the cancer stages differ in some respects compared to the general oral cancer population. Second, due to the small sample size, the robustness of the statistical analysis may be unstable. Third, this study did not examine reliability and validity, including predictive validity. Data analysis on a larger-scale sample will be required for future research.

## 5. Conclusions

The incidence of postoperative oral dysfunction tends to be highest in Type 2, and POD-10 is a reliable and validated instrument for assessing postoperative oral dysfunction in patients with oral cancer.

## Figures and Tables

**Table 1:** Definition of the Matsuda-Kanno classification and cut-off values for each oral function measurement.

Type	Name	Definition	Reference Values for Diagnostic Criteria
I	Transport type	A condition in which dysfunction occurs during the oral preparatory and transit phases of swallowing due to treatment-induced damage to the tongue, palate, buccal mucosa, or oral floor.	Masticatory function (cut-off value: 83 mg/dL) EAT-10 (cut-off value: 12) Tongue pressure (cut-off value: 14 kPa)
II	Occlusion type	Conditions in which occlusion is impaired due to the loss of maxilla and mandibular or teeth from treatment.	Occlusal force (cut-off value: 230 N)
III	Oral hygiene type	Conditions in which the self-cleaning and antibacterial moisturizing functions of the oral cavity are impaired by treatment.	Number of microorganisms (cut-off value: 10^6.5^ or more) Oral dryness (cut-off value: 27.0) Chief complaint of subjective oral health perception

**Table 2:** Descriptive analysis of patient characteristics (N = 62).

Item	Category	n (%) or Median (IQR)
Age (years)		71.0 (63.0–76.5)
Sex	Male	42 (67.7)
	Female	20 (32.3)
BMI		19.9 (18.1–23.4)
Brinkman index		0.0 (0.0–440.0)
Alcohol consumption	None	29 (46.8)
	Social drinker	5 (8.1)
	Regular drinker	28 (45.2)
Performance status	0	46 (74.2)
	1	9 (14.5)
	2	1 (1.6)
	3	6 (9.7)
Primary tumor site	Tongue	25 (40.3)
	Maxillary gingiva	12 (19.4)
	Mandibular gingiva	12 (19.4)
	Palate	3 (4.8)
	Oral floor	5 (8.1)
	Buccal mucosa	2 (3.2)
	Central mandible	2 (3.2)
	Lower lip	1 (1.6)
Tumor stage	I	12 (19.4)
	II	8 (12.9)
	III	11 (17.7)
	IV	31 (50.0)
Treatment	Surgery	25 (40.3)
	Surgery + RT	10 (16.1)
	Surgery + CT	4 (6.5)
	Surgery + CRT	23 (37.1)
Neck dissection (yes)		42 (67.7)
Reconstruction (yes)		40 (64.5)
Number of teeth		17.0 (0.0–25.0)
Oral function measurement	Microorganisms (Grade)	4.0 (2.0–5.0)
	Oral dryness	24.6 (21.2–26.7)
	Occlusal force (N)	270.4 (27.8–458.6)
	Tongue pressure (kPa)	15.7 (5.3–25.0)
	Masticatory function (mg/dL)	58.0 (12.0–159.0)
	EAT-10	16.5 (5.5–25.3)
FOIS		5.0 (5.0–6.0)
RSST		3.0 (3.0–4.0)
MNA-SF	Normal nutritional status	13 (21.0)
	At the risk of malnutrition	25 (40.3)
	Malnourished	24 (38.7)
Postoperative oral dysfunction	None	17 (27.4)
	Type I	3 (4.8)
	Type II	13 (21.0)
	Type III	11 (17.7)
	Type I & II	1 (1.6)
	Type II & III	7 (11.3)
	Type I & III	6 (9.7)
	Type I & II & III	4 (6.5)

IQR, interquartile range; BMI, body mass index; RT, radiotherapy; CT, chemotherapy; CRT, chemoradiotherapy; FOIS, functional oral intake scale; RSST, repetitive saliva swallowing test; MNA-SF, Mini Nutritional Assessment-Short Form.

**Table 3:** Descriptive analysis of POD-10.

Question	Median (IQR) or Mean (SD)
Q1. It takes a long time to eat because of mouth problems.	2.0 (1.0–3.0)
Q2. There are food restrictions due to mouth problems.	2.5 (1.0–4.0)
Q3. Mouth problems have become a barrier to going out to eat.	2.0 (1.0–3.0)
Q4. It takes extra effort to chew solid food.	2.0 (1.0–3.0)
Q5. Food will remain in your mouth.	2.0 (0.0–3.0)
Q6. Food or saliva spills from your mouth.	1.0 (0.0–3.0)
Q7. I feel my mouth getting dry.	2.0 (0.0–3.0)
Q8. I find it painful to talk	2.0 (0.0–2.0)
Q9. Due to mouth problems, the other person cannot understand your words.	1.0 (0.0–2.0)
Q10. I am not confident about the appearance of my mouth.	1.0 (0.0–2.3)
Total score	18.0 (10.8)

SD, standard deviation; IQR, interquartile range. This English version of POD-10 has not been validated.

## Data Availability

All data were collected from this prospective cohort study. The derived data supporting the findings of this study are not available because this study is ongoing.
